# Recruitment of cyanobacteria by reverse transcription quantitative real-time PCR based on expression of *Microcystis* gene

**DOI:** 10.7717/peerj.7188

**Published:** 2019-06-26

**Authors:** Long Yu, Xiaofei Wu, Yang Yu, Limei Shi, Min Zhang

**Affiliations:** 1 College of Biotechnology and Pharmaceutical Engineering, Nanjing University of Technology, Nanjing, China; 2 State Key Laboratory of Lake Science and Environment, Nanjing Institute of Geography & Limnology, Chinese Academy of Sciences, Nanjing, China

**Keywords:** *espL*, *mcyA*, *gvpC*, qRT-PCR, Cyanobacterial recruitment, Relative expression, Algal bloom

## Abstract

In this study, a SYBR Green quantitative real-time PCR method was established and applied. Relative expression of the synthetic genes from *Microcystis* gas vesicles (*gvpC*), algal toxin genes (*mcyA*), and polysaccharides (*espL*) from water and sediments of Meiliang Bay and from the center of Lake Taihu were tested from January to June, 2017. Indoor *Microcystis aeruginosa* was used as the control group. The kit for total RNA extraction in *Microcystis* was optimized. Results showed that the optimized kit extracted high-concentrations and high-quality total RNA from *Microcystis*. The extraction purity and concentration were significantly higher than those extracted by the original kit. The transcription level of *gvpC* increased gradually until a peak was reached in March. However, expression of *gvpC* decreased continuously at the proliferating and floating stages of Cyanobacterial biomass. The maximum level of expression of *gvpC* in April in comparison to expression of *mcyA* in March occurred first. We found that the SYBR Green qRT-PCR method, which is characterized by high specificity, repeatability, is rapid, and can be used for quantitative detection of expression of *gvpC*, *mcyA*, and *espL*. The recruitment of cyanobacteria is the process in which cyanobacteria in the sediment began to regain their activity, started to grow and migrated to the water column.

## Introduction

Quantitative real-time polymerase chain reaction (qRT-PCR) employs a fluorescent substance during the PCR process. With its high sensitivity, specificity and efficiency, its simple operation, without any requirements for pre-amplification, qRT-PCR can amplify multiple target genetic molecules in the same reaction system under different specific primer conditions (Freeman, Walker & Vranna, 1999). The fluorescence signals increase proportionally as the PCR progresses so that the quantity of specific products is determined by continuously detecting the change of the fluorescent signal, and therefore the initial template quantity of the target gene can be deduced (Sonawane & Tripathi, 2013). SYBR Green only emits extremely faint fluorescence in the free state. However, it can bond with double-stranded DNA, enhancing its fluorescence intensity by more than 1,000 times. Due to its low cost, SYBR Green is applicable to different templates; it does not involve a specificity probe rather the specificity relies on the specificity of the primers. Nevertheless, all double-stranded DNA can generate fluorescence, and quantitative analysis, and melting curve analysis of the target genes (Nadkarni et al., 2002).

As the third largest freshwater Lake in China, Lake Taihu is typical of large Lakes and suffers occasional cyanobacterial blooms. Studies have analyzed the chemical, physical, and biological causes of cyanobacterial blooms and elaborated possible causes, especially in the case of *Microcystis*, the dominant species in algal blooms (Liu et al., 2012). Macroscopic studies, based on remote sensing and telemetry of the formation mechanism of cyanobacterial blooms, have observed cyanobacteria in the whole Lake, while microscopic studies have a tendency to analyze the physiological processes of cyanobacteria using molecular biology methods (Wu et al., 2008). The recruitment of cyanobacteria has been studied both macroscopically and microscopically in addition to using molecular biology techniques.

The floating of *Microcystis* is the key stage of cyanobacterial bloom and the gas vesicle is considered a major factor providing buoyancy for cyanobacterial blooms. Exopolysaccharides (EPS), which are released by *Microcystis* cells, is the main component of the sheath of *Microcystis*. The sheath plays an important role in the *Microcystis* floating during the recruitment. When studying the dynamic changes of key genes in the microbial community, DNA is used as the target gene sequence of template amplification and is often influenced by dying cells or free DNA residues, which makes it difficult to determine its ecological influence (Miskin, Farrimond & Head, 1999). Targeting RNA sequence can be used to analyze the active organisms within a microbial community, the analysis of mRNA expression in particular can offer more information about specific active microorganisms. Currently, qRT-PCR can detect RNA at very low levels and target genes are amplified and quantified (Jing et al., 2008). Here, specific primers are designed based on the key factors of cyanobacterial recruitment, which can study gas vesicles, algal toxins and EPS and detect the dynamic changes in the quantity and buoyancy of cyanobacteria in different external environments. Results will provide important data to understand the resuscitation of cyanobacteria and protect water bodies from cyanobacterial blooms.

In this study, an RNA kit was optimized for extraction of cyanobacteria in water and sediment samples from Lake Taihu. A quantitative real time PCR (qRT-PCR) was established by using the QuantiFast SYBR Green PCR Master Mix to detect relative expression of *gvpC*, *mcyA*, and *espL*, which proved beneficial in the study of the recruitment of cyanobacteria.

## Materials and Methods

### Sampling points and sample collection

**Figure 1:**
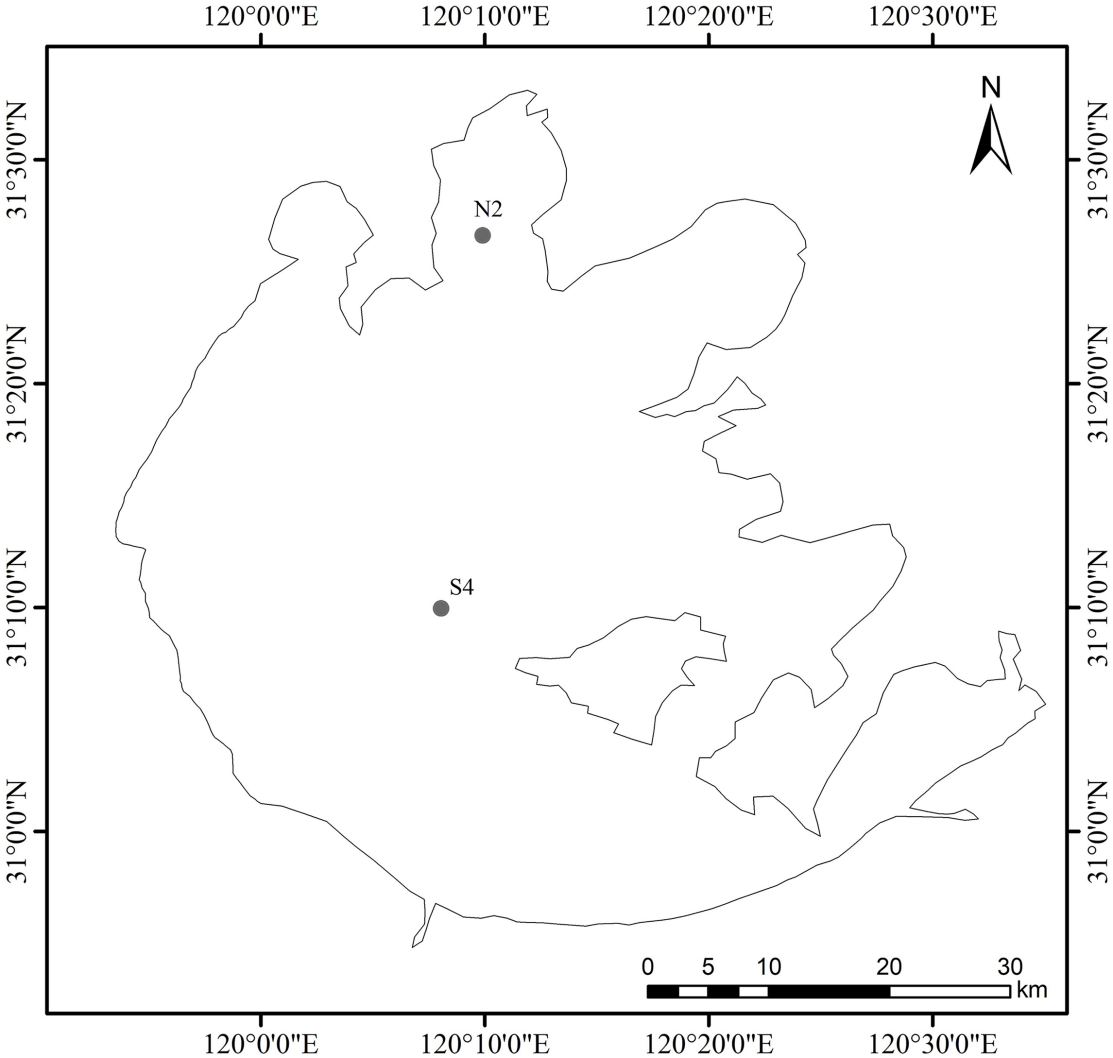
Sampling sites in Meiliang Bay and the center of Lake Taihu. In this study, two sampling points were designated in Lake Taihu site: N2 was located at Meiliang Bay (N2) (31°25′53.4″N, 120°12′42.5″E) and site S4 was located in the center of the lake (31°23′2.76″N, 120°18′3.6″E).

Cyanobacterial blooms in Lake Taihu are initially formed in specific regions during spring and summer and then spread over the whole Lake. Meiliang Bay, in the north of Lake Taihu, is one such region and has the most serious eutrophication. In recent years, Meiliang Bay is one of the earliest areas to experience cyanobacteria blooms (April–May). Compared with Meiliang Bay, the concentration of nutrients in the rest of the Lake is low. The center of the Lake is predominantly covered by hard sediments and cyanobacterial blooms has never occurred across the whole Lake before. In this study, two sampling points were chosen in Lake Taihu. Site N2 was located at Meiliang Bay and site S4 was located in the center of the lake. These two sampling points are shown in Fig. 1. Samples were collected every month from January to June, 2017 within ten days of the middle of each month. Water samples were stored in 2.5 m long PVC tubes and mixed evenly before RNA extraction. Water samples (150 ml) were filtered by GF/C membrane to extract RNA, the GF/C filter membrane was used at a temperature below −80 °C according to the Qiagen RNeasy Mini Kit instructions for RNA extraction before RNA*later*™ was added. Sediment samples were collected by a column sampler with an inner diameter of 62 mm and the surface sediments (0–3 cm thickness) were mixed evenly and then transferred into a sterile plastic box. Sediment samples (1 g) were put in a 2 ml centrifuge tube without RNA enzyme, and 1 ml RNA*later*™ added and mixed evenly. Samples were stored below −80 °C until RNA extraction.

### Main apparatus and reagents

Instruments: QuantStudio™ 7 Flex Real-Time PCR System, ChemiDoc MP (BIO-RAD, Hercules, CA, USA), MP FastPrep-24 high-speed, digitally controlled benchtop homogenizer, NanoDrop 2000/2000c micro-ultraviolet spectrometer.

Kits and reagents: Qiagen RNeasy Mini Kit, OMEGA Soil RNA Kit, RNase-free DNase I kit (TaKaRa, Kusatsu, Japan), Transcriptor First Strand cDNA Synthesis Kit, QuantiFast SYBR Green PCR Master Mix, absolute ethyl alcohol, DEPC H_2_O, phenol:chloroform:isoamylol=25:24:1, and β-ME. The rest of the reagents were all analytical pure reagents.

### Primer design and synthesis

Primer sequences are listed in Table 1. The 16SrRNA of *Microcystis* was selected as the reference gene. Primers of the target genes *gvpC*, *mcyA*, *espL* were synthesized by Shanghai Sangon Bioengineering Company (Shanghai, China).

**Table 1:** Primer sequences.

Genes	Primer	Sequences (5′-3′)	References
16srRNA	Micro184F	GCCGCRAGGTGAAAMCTAA	Rinta-Kanto et al. (2005)
	Micro431R	AATCCAAARACCTTCCTCCC	
*gvpC*	*gvpC*-F	TGCTTTGCGTCAGTCTTTCC	Mlouka et al. (2004)
	*gvpC*-R	TCCTTCACCTGTTTGGCTCT	
*mcyA*	*mcyA*-F	GGCTGCGTAGCGGTTTCCTT	Oh, Han & Cho (2010)
	*mcyA*-R	CGGCTTTATCTCCTCAACAGTCTC	
*espL*	*espL*-F	CGATGGGTGCGTTATCTTCC	Gan et al. (2012)
	*espL*-R	GCCGATTACTGGCTGTCCTG	

**Note:**

Primer sequences are listed in the table. The specificity 16srRNA of *Microcystis* was selected as the reference gene. Primers of target genes like *gvpC*, *mcyA*, *espL* were synthesized by Shanghai Sangon Bioengineering Company.

#### Optimisation of extraction method

##### Optimisation of total RNA extraction kit in water samples

The Qiagen RNeasy Mini Kit for RNA extraction was modified according to the instructions. The RNA extraction purity and concentration were tested by several experiments to determine the validity of the optimization steps. RNase-free water was used as the blank control. RNA concentration and purity (OD260/280) in the five μl RNA samples were tested by NanoDrop 2000/2000c micro-ultraviolet spectrometer.

##### Optimization of total RNA extraction kit in sediment samples

The OMEGA Soil RNA Kit was modified according to the instructions. The RNA extraction purity and concentration were tested by several experiments to determine the validity of the optimization steps. RNase-free water was used as the blank control. RNA concentration and purity (OD260/280) in the five μl RNA samples were tested by NanoDrop 2000/2000c micro-ultraviolet spectrometer.

##### Reverse transcription reaction and qRT-PCR

The reverse transcription was implemented in a 20 μl reaction system using the Transcriptor First Strand cDNA Synthesis Kit. The reaction system was composed of one μl Anchored-oligo (dT), two μl random hexamer primer, 400 ng RNA and appropriate PCR water, four μl transcriptor reaction buffer solution, 0.5 μl RNA inhibitor, two μl deoxynucleotide mixture, and 0.5 μl transcriptor reverse transcriptase. The inverse transcription parameters were 10 min at 25 °C, 30 min at 55 °C and 5 min at 85 °C. After the incubation, the resulting cDNA was placed on ice for immediate use or else stored below −20 °C for long-term storage.

The qRT-RCR reaction system totaled 25 μl, including 12.5 μl 2× QuantiFast SYBR Green PCR Master Mix, two μm primer, one μl template, and 10.5 μl RNAse-free water. The cDNA template which used 16SrRNA as the primer, was diluted by a factor of 10 and other cDNA templates remained undiluted. The PCR reaction was performed on the QuantStudio™ 7 qRT-PCR instrument. The PCR process was comprised of 3 min of initial denaturation at 95 °C, 15 s of denaturation at 95 °C, 30 s of annealing at 60 °C, 45 s of extension at 72 °C, for 40 cycles, followed by a melt curve stage 15 s at 95 °C, 60 s at 60 °C, and 15 s at 95 °C. An automatic analysis was accomplished in QuantStudio™ Design & Analysis Software v1.3.1 after the PCR to gain the corresponding threshold cycle values (Ct). Ct is defined as the number of cycles before fluorescence signal crosses the threshold or background level. Quantification of target gene levels was performed by the QuantStudio™ Design & Analysis Software v1.3.1 using the standard curve. Gene expression was assessed by the Ct of the reaction. During the PCR process, 16SrRNA was used as the reference gene to correct the copy number of cells of the PCR template, thus eliminating the addition of the intergroup sample.

*Microcystis aeruginosa* 7,806 was cultured in the laboratory and used as the control group, while BG-11 was used as the culture medium. The culture temperature, illumination intensity, and illumination period were 25 °C, 40 μE·(m^2^·s)^−1^ and 12 h:12 h, respectively. Samples were collected before *Microcystis* cells entered the logarithmic phase, and centrifuged before RNA extraction. Extracted samples were used as the reference sample. To prove the consistency of amplification efficiency between the target genes (*gvpC*, *mcyA*, and *espL*) and reference gene (16SrRNA), cDNA templates of *M. aeruginosa* 7,806 were diluted into a series of gradients. All diluted samples were amplified by 16S and target genes (*gvpC*, *mcyA*, and *espL*). The curve of ΔCt (ΔCt = Ct_target gene_ − Ct_16S_) relative to the log value of cDNA concentration gradients was drawn. The same amplification efficiency was indicated when the absolute value of the slope was close to zero (Livak & Schmittgen, 2001).

The amplification curve of qRT-PCR was divided into three stages: fluorescenct background signal stage, exponential amplification stage of fluorescenct signals and plateau stage. Ct value could be deduced directly after quantitative PCR. Higher Ct values implied smaller copy numbers of target genes. Specificity of reaction products was analyzed by the amplification curve and melt curve (80–90 °C).

##### Data processing and analysis

The relative expression of genes was expressed by 2^−ΔΔCt^ (XiuMin et al., 2007). The calculation formula of ΔΔCt was as follows:

$${\rm{\Delta \Delta Ct }} = {\rm{ }}{\left( {{\rm{C}}{{\rm{t}}_{{\rm{target\ gene}}}} - {\rm{C}}{{\rm{t}}_{16S}}} \right)_{{\rm{stress}}}} - {\left( {{\rm{C}}{{\rm{t}}_{{\rm{target\ gene}}}} - {\rm{C}}{{\rm{t}}_{16S}}} \right)_{{\rm{control}}}}$$

where Ct_target gene_ is the Ct value of a target gene, Ct_16S_ is the Ct value of 16S rRNA, stress is the Ct of the test gene, and control is the Ct value of the laboratory control gene.

The intergroup difference was analyzed by one-way analysis of variance with *p* < 0.05 considered a significant difference. A *p*-value <0.01 represented an extremely significant difference.

Data processing and analysis were performed by WPS Office 2016, SPSS 20.0, and Canoco 4.5.

## Results

### Optimization of total RNA extraction methods in water sample and sediment sample

Optimization steps of total RNA are shown in Table 2.

**Table 2:** Optimization steps of total RNA.

Optimization steps of total RNA extraction method in water samples	Optimization steps of total RNA extraction method in sediment samples
1. Samples were transferred into a purple Lysing Matrix E (with glass beads of different diameters: 1.2, 0.074, and 4 mm). The mixture of 600 μl Buffer RLT and six μl β-ME was added and mixed evenly by up-down repeatedly.	1. Samples were transferred into a blue Lysing Matrix B (with a glass bead of 0.1 mm diameter). The mixture of 400 μl Buffer SRX and 40 μl HTR2 Reagent was added into each Lysing Matrix E and mixed evenly by up-down repeatedly.
2. The Lysing Matrix B was placed in the Fast Prep-24 sample processor and parameters set at six m/s, 30 s, four times. The matrix was crushed and then immediately placed on a piece of ice. In this process, algal cells were split completely.	
3. During RNA extraction, RNase-free DNase I(TaKaRa, Kusatsu, Japan) kit used the DNase to eliminate polluted DNA.	3. The optimized OMEGA Soil RNA Kit eliminated DNA by using the HiRind DNA Mini column.
4. Finally, elution based on RNase-free water preheated to 65 °C was performed twice to increase the extraction amount of RNA in blue algae.	4. Preheated DEPC Water (50 μl) was added twice.

**Note:**

Optimization of total RNA extraction methods in water sample and sediment sample. The RNA extraction concentration and purity increased dramatically after the optimization.

**Figure 2:**
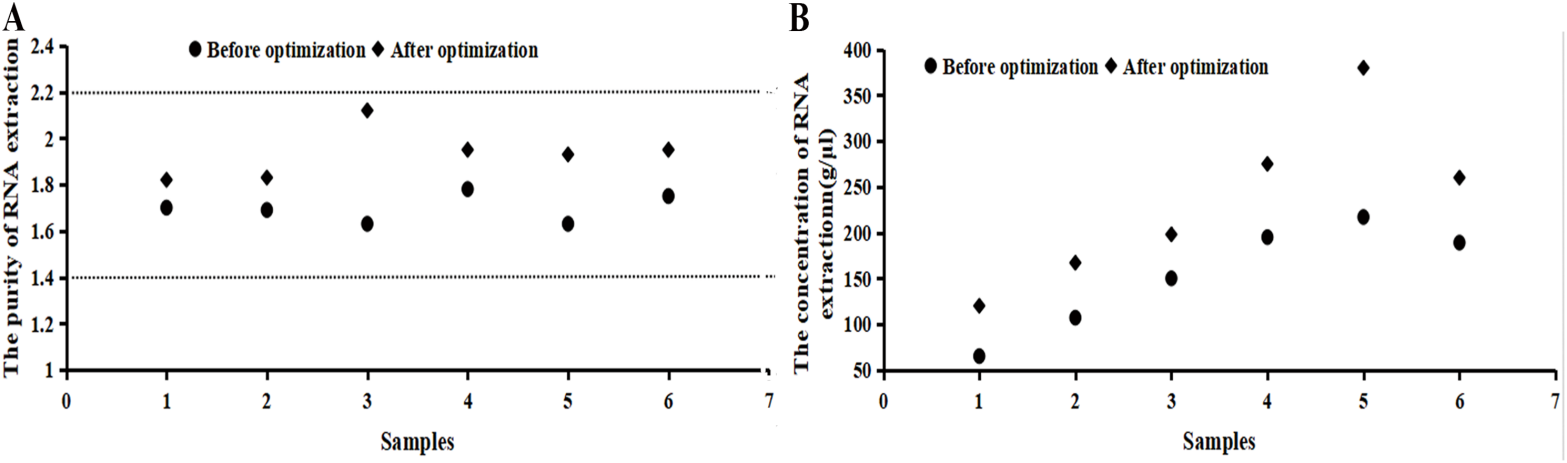
Purity and concentration after optimization basing on the RNA samples. (A) The purity of RNA extraction. (B) The concentration of RNA extraction. It can be seen from the image that the numerical value of purity before the optimization was between 1.5 and 1.8, which increased to 1.8–2.2 after the optimization. The RNA extraction concentration increased dramatically after the optimization. RNA concentration and purity (OD260/280) were measured by a NanoDrop 2000/2000c micro-ultraviolet spectrometer, indicating that the extracted RNA was applicable for a follow-up experiment.

It can be seen from Fig. 2 that the numerical value of purity before the optimization was between 1.5 and 1.8, which increased to 1.8–2.2 after the optimization. The RNA extraction concentration increased dramatically after the optimization. If samples did not fall within the acceptable range, the 260/230 ratio was checked or samples were taken again.

### Test results of RNA extraction and purity in *Microcystis*

RNA concentration and purity (OD260/280) were measured by a NanoDrop 2000/2000c micro-ultraviolet spectrometer. OD260/280 ranged from 1.8 to 2.2, indicating that the extracted RNA met the requirement of purity and was able to be used in follow-up experiments. The ΔCt curve, relative to the log value of cDNA concentration gradients, was drawn after the amplification of the target genes *gvpC*, *mcyA*, and *espL* by diluted cDNA. The linear equations were *y* = −0.0908*x* + 10.155, *y* = −0.0177*x* + 10.059 and *y* = −0.0502*x* + 10.088. The absolute slopes of the two straight lines were close to zero, indicating the consistent amplification efficiency between target genes (*gvpC*, *mcyA*, and *espL*) and the reference gene.

### Amplification specificity test of qRT-PCR

**Figure 3:**
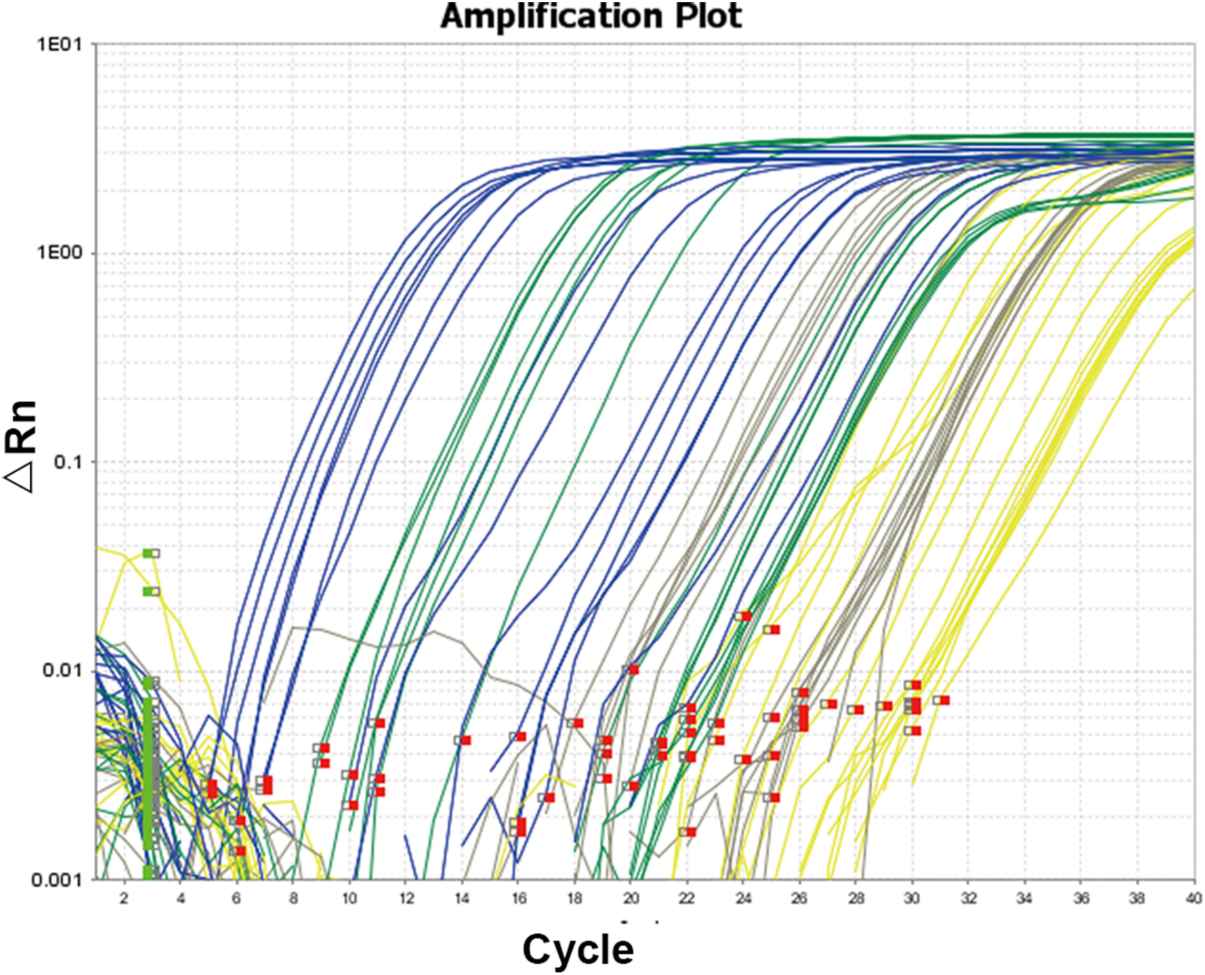
Amplification curve of quantitative real-time PCR. The amplification curve and melt curve of qRT-PCR are shown in the image. The amplification curve of qRT-PCR was divided into the fluorescence background signal stage, the exponential amplification stage of fluorescence signals and the plateau stage. Ct values could be deduced directly after qRT-PCR. Higher Ct values implied smaller copy numbers of target genes.

**Figure 4:**
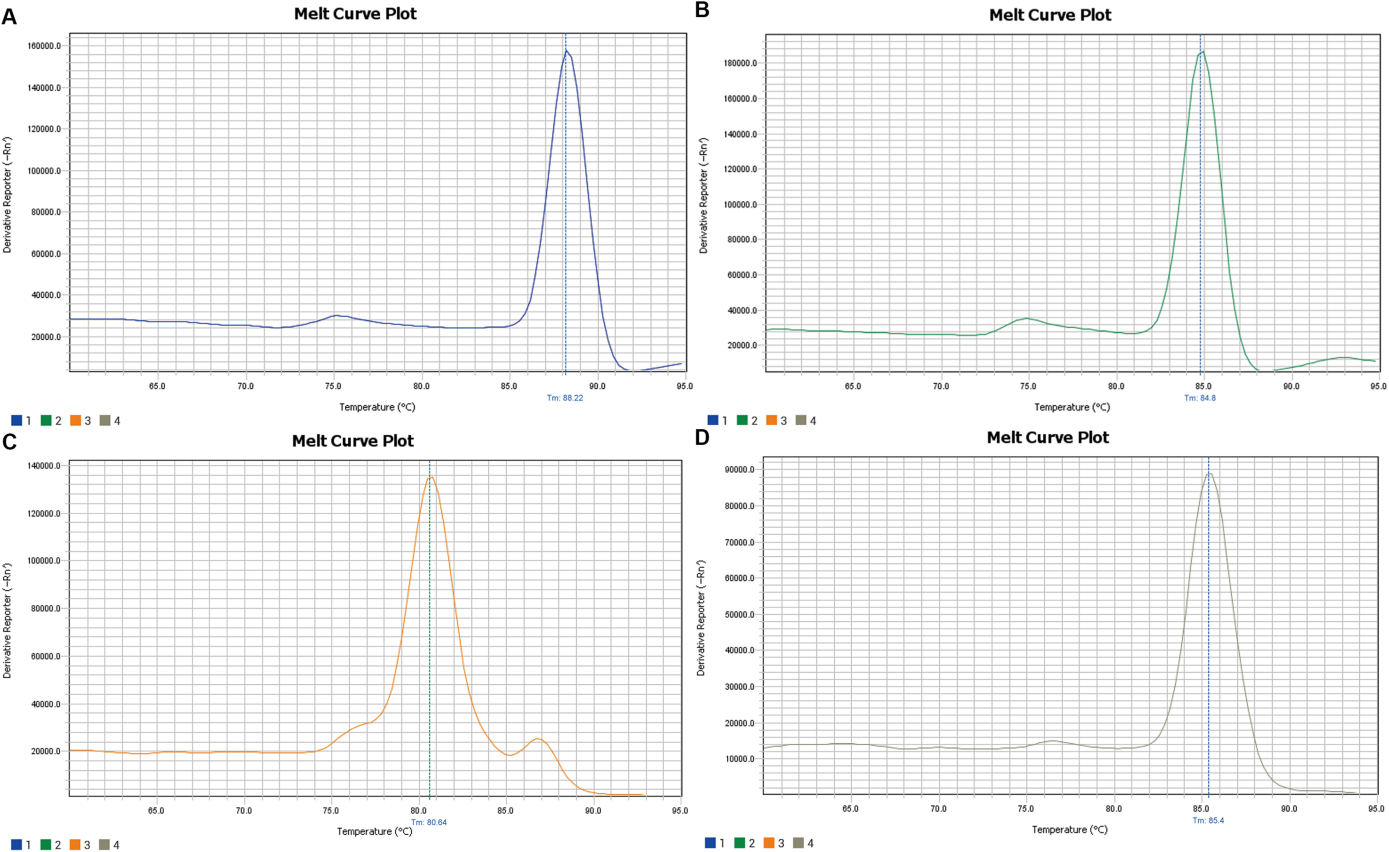
Melting curve of *16srRNA* (A), *gvpC* (B), *mcyA* (C), *espL* (D). It can be seen from the melt curve that the amplification curves of *16srRNA*, *gvpC*, *mcyA*, *espL* of *Microcystis* had only one peak and *T*_m_ between 80 and 90 °C, reflecting only one amplification product and the amplification primer had strong specificity.

The amplification curve and melt curve of qRT-PCR are shown in Figs. 3 and 4. The amplification curve of qRT-PCR was divided into the fluorescence background signal stage, the exponential amplification stage of fluorescence signals, and the plateau stage. Ct values could be deduced directly after qRT-PCR. Higher Ct values implied a smaller number of target gene copies. It can be seen from the melt curve that the amplification curves of three target genes of *Microcystis* had only one peak and that the *T*_m_ was between 80 and 90 °C, reflecting only one amplification product and that the amplification primer had a strong specificity.

### Monthly trends of relative expression of *gvpC*, *mcyA*, and *espL* and phycocyanobilin concentration in water at N2 and S4

**Figure 5:**
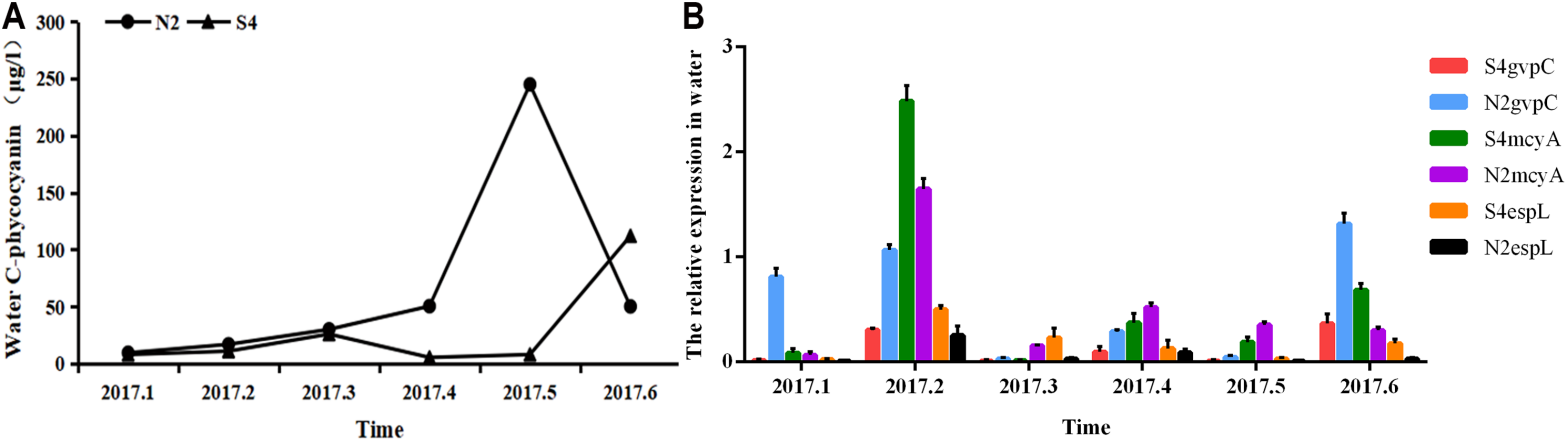
Phycocyanobilin concentration (A) and Relative expression of *gvpC*, *mcyA*, *espL* in RNA of cyanobacteria in water (B). Each data point indicates that Phycocyanobilin concentration in water has a trend at S4 and N2. Histogram indicates the relative expression of *gvpC*, *mcyA*, *espL* in RNA of cyanobacteria at S4 and N2 in water.

It can be seen from Fig. 5 that in water, phycocyanobilin concentrations at N2 increased slowly from January to April, increased rapidly from April to May, before dropping sharply after May. The phycocyanobilin concentration at S4 increased slowly from January to March, decreased slowly from March to April, and then increased quickly after May.

The relative expression of *gvpC* at N2 rose quickly from January to March and reached a maximum value of 2.4828, but then dropped sharply after March. The relative expression of *gvpC* at S4 increased quickly from January to February and reached a maximum value of 0.8120. Subsequently, the relative expression of *gvpC* at S4 declined slowly.

The relative expression of *mcyA* at N2 grew sharply from January to April and peaked at 0.5205, subsequently dropping significantly. The relative expression of *mcyA* at S4 first increased before decreasing from January to March. It then increased quickly from March to May and reached a maximum value of 0.2309. It decreased steadily after May.

The relative expression of *espL* at N2 increased quickly from January to April and reached a peak of 1.3165. It dropped quickly from February to April before declining slowly after April. The relative expression of *espL* at S4 increased slowly from January to April. It rose rapidly from February to April and reached a maximum of 0.3539 and then subsequently declined. The OD260/280 of all samples of RNA ranged between 1.8 and 2.2, indicating that the extraction purity of RNA was adequate. As shown by the one-way analysis of variance test, there was a significant difference in the relative expression of *gvpC* between water samples at N2 and S4 (*p* < 0.05).

To summarize, phycocyanobilin concentrations at N2 increased gradually from January to May and reached a maximum at level May. The maximum phycocyanobilin concentration was maintained through the winter and only began to decrease after the formation of the algal bloom. The transcription level of *gvpC* increased gradually and reached its peak level in March. However, the expression level of *gvpC* decreased gradually in the floating stage of the cyanobacteria biomass. This implies that the expression of *gvpC* and the formation of gas vesicles seem to be a landmark change in the ability of *Microcystis* to get rid of dormancy and regain buoyancy. When cyanobacterial cells recovery to normal cells, continuous expression of *gvpC* is not required. The relative expression of *mcyA* differed from the expression of *gvpC*, in that it increased from January to April and reached a maximum in April. The relative expression of *espL* increased quickly from January to February and then decreased steadily after February.

The phycocyanobilin concentration at S4 first increased before decreasing slowly from January to June. The maximum value was achieved in June and was maintained through the whole winter. The transcription level of *gvpC* increased gradually and reached a maximum in February. However, the expression level of *gvpC* decreased gradually in the growth and floating stages of the cyanobacteria biomass. The relative expression of *mcyA* differed from the expression of *gvpC*, and increased from January to May and reached a maximum in May. The relative expression of *espL* increased rapidly from January to April and then decreased steadily after April.

### Monthly trends of relative expression of *gvpC*, *mcyA*, and *espL* and phycocyanobilin concentration in sediments at N2 and S4

**Figure 6:**
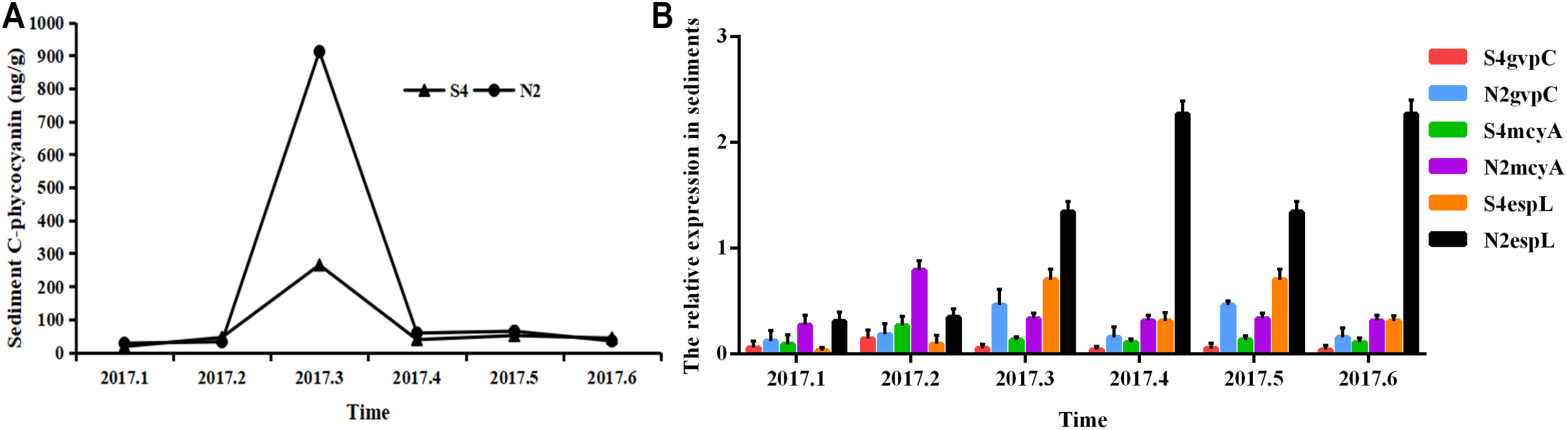
Phycocyanobilin concentration (A) and relative expression of *gvpC*, *mcyA*, *espL* in RNA of cyanobacteria in sediments (B). Each data point indicates that Phycocyanobilin concentration in sediments has a trend at S4 and N2. Histogram indicates the relative expression of *gvpC*, *mcyA*, *espL* in RNA of cyanobacteria at S4 and N2 in sediments.

It can be seen from Fig. 6 that in sediments, the phycocyanobilin concentration at N2 increased rapidly from February to March, but then dropped sharply from March to April. The reduction rate then decreased after April. The phycocyanobilin concentration at S4 increased quickly from February to March, decreased rapidly from March to April, and then decreased slowly after April.

The relative expression of *gvpC* at N2 rose quickly from January to March and reached the maximum of 0.4641, but then dropped sharply from March to April and decreased slowly after April. The relative expression of *gvpC* at S4 increased quickly from January to February and reached the maximum of 0.1454. Subsequently, the relative expression of *gvpC* at S4 began to decline quickly from February to March and then slowly after March.

The relative expression of *mcyA* at N2 achieved a sharp growth from January to February and reached a peak of 0.7930 before dropping significantly after February. The relative expression of *mcyA* at S4 increased quickly from January to February and reached the maximum of 0.2685 and then decreased after February.

The relative expression of *espL* at N2 increased slowly from January to February, but increased dramatically and reached a peak of 2.2703 from February to April. It then decreased from April. The relative expression of *espL* at S4 increased slowly from January to February, rose quickly from February to March and reached a maximum of 0.7045. It declined rapidly from March and May before decreasing more slowly after May.

To summarize, the phycocyanobilin concentration at N2 increased gradually from January to March and reached a maximum in March before decreasing significantly after March. The transcription level of *gvpC* increased gradually and reached a peak in March. The relative expression of *mcyA* increased from January to February and reached a maximum in February which differed from *gvpC* expression. Synthesis of algal toxins is directly related to the reactivation of *Microcystis* in sediments and is conducive to the formation of the *Microcystis* community. The relative expression of *espL* increased rapidly from January to April before decreasing steadily after April.

The phycocyanobilin concentration at S4 increased gradually between January and March with a maximum value in March before beginning to decrease after March. The transcription level of *gvpC* increased gradually and reached a maximum in February. The relative expression of *mcyA* increased from January to February and reached a maximum in February, which differed from the expression of *gvpC*. Synthesis of algal toxins is directly related to the reactivation of *Microcystis* in sediments and is conducive to the formation of the *Microcystis* community. The relative expression of *espL* increased rapidly from January to March and then decreased steadily after March.

## Discussion

Shallow Lakes are not only an important natural landscape, but also provide people with tourism, maintaining biodiversity, water supply, aquaculture, water conservancy, flood control, and climate regulation. They are one of the most important water resources on the earth and play an inestimable role on social and economic development in that region. However, with the rapid development of economy and society, the problem of cyanobacterial blooms is becoming more and more serious. In order to solve these problems, we should first make theoretical innovation and it is important to make clear the process on recruitment of cyanobacteria, so as to provide the evidence base to guide solving the problem for the water environment. In this study, synthetic primers of *gvpC*, *mcyA*, and *espL* were designed. After adjusting the RT-PCR reaction system and conditions several times, the primers were optimized to the same PCR reaction conditions.

The relative expression of *gvpC*, *mcyA*, and *espL* were detected in Lake Taihu between January and June, 2017. Dynamic changes in the quantity and buoyancy of cyanobacterium different external environments were tested at the level of transcription. RT-PCR has high sensitivity, high specificity and high accuracy, facilitating qualitative but also quantitative changes in the composition and quantity of microbial populations in the field. Therefore, in the process of studying the recruitment of cyanobacteria, it has theoretical and practical significance for predicting cyanobacterial blooms and taking timely measures to slow down the ecological disasters of cyanobacterial blooms.

It is difficult to distinguish living from dead algae cells using traditional analytical approaches. Morphological features of algal gas vesicles, microcystic toxins, and quantitative real-time PCR of DNA, fail to reflect the states of active algae which live through the winter. Our proposed methodology however, applies relative quantitative qRT-PCR to enable comparison of gene expression differences between field samples collected at different times and laboratory cultured microcystic samples based on RNA extraction.

Moreover, temporal and spatial dynamic changes of cyanobacteria in water and sediments samples were tested using qRT-PCR to analyze multiple samples simultaneously, and is insensitive to suspended particulates and the state of algal community state in complicated environments. The proposed method shows high repeatability and accuracy and can accomplish quantitative detection of samples in a short time. Quantitative detection of mRNA expression based on Northern Blotting or RNase Protection Assay has been reported previously by other studies (Vrydag & Michel, 2007). However, qRT-PCR is known to be more sensitive and requires fewer less volume of sample RNAs and provides sequence information compared to those technologies mentioned. There are abundant studies on the quantification of synthetase genes in microcystic toxins at the DNA level, but only few have discussed these at the RNA level (Stelzer, Loftin & Struffolino, 2013). Hence, recovering rRNA sequences from RNA templates using RT-PCR implies that the biological source is active at sampling and is a good indicator of active cells.

The RNA extraction process generally crushes cells through the need to grind them in order to eliminate polysaccharide, protein, and DNA contamination. Samples are further washed and precipitated to get a high-purity RNA sample. Effective cell lysis is the key to high-quality and high-yield RNA extraction (Liang et al., 2013; Yang et al., 2013). In this study, algal cells were placed in lysing tubes and crushed fully by an MP FastPrep-24 nucleic acid isolation machine (USA). Next, the solution was transferred to RNease Mini Spin Columns for full-speed centrifugation in order to eliminate contamination of nucleic acids by polysaccharides and proteins in cells. DNA contamination in RNA was eliminated by RNase-free DNase I (TaKaRa, Kusatsu, Japan) kit with DNase. RNA was obtained after optimization with the appropriate purity and high concentration. This simple extraction method of RNA avoids the potential harm of toxic reagents, shortens the experimental time, and reduces the cost.

The life history of cyanobacteria generally has two independent and dependent stages: one in the water column and one in the sediment. Generally speaking, the quantity of active cyanobacteria in sediments during the overwintering period determines the reactivation quantity in the next year and is one of the direct causes of algal blooms. Reactivation is the key process that allows cyanobacterial blooms. Cyanobacteria cells adjust their vertical distribution in the water column through the breaking and synthesis of *gvpC* (Kinsman, Ibelings & Walsby, 1991). Cells can float to the water surface by synthesis of gas vesicles, increasing illumination and CO_2_ supply (Walsby et al., 2010).

Light not only provides energy for synthesis of the gas vesicles, but can also serve as an environmental signal to regulate the synthesis of gas vesicles (Walsby, 1998). Given inadequate nutritive salts on the surface layer of a Lake, the cyanobacteria will sink into deeper water layers by reducing the synthesis of gas vesicles to absorb enough nutritive salts (Smayda, 1997). The reactivation process involves the transformation of *Microcystis* from a monocell or microcommunity into a large community and a floating *Microcystis* community which can migrate vertically by adjusting its buoyancy. Thus, vertical migration of algae is sensitive to community size and large communities migrate more quickly than smaller ones (Kromkamp & Walsby, 1990).

Additionally, *Microcystis* toxins play an important role in bloom outbursts and dominance (Benndorf, Ihle & Jähnichen, 2005). *Microcystis* toxins might participate in intracellular signal transmission and gene control (Dittmann et al., 2001). Recently, it was reported that the synthesis of algal toxins is directly correlated with the reactivation quantity of *Microcystis* in sediments and is conducive to the formation of an algal community (Cao et al., 2005). The sheaths of *Microcystis* also make a significant contribution to flotation on recruitment. EPS is released by *Microcystis* cells as the main component of the sheaths of a *Microcystis* community. In the recruitment of cyanobacteria in spring, a large number of cells that have died or are still dormant will affect the judgment of recruitment cells significantly. More importantly, recruited cyanobacteria entered the water during field investigations and it was very difficult in the determination of the quantity of recruitment cells in the water column and sediments. Hence, a scientific judgment on whether the *Microcystis* reactivation is initiative or passive could be determined by detecting the transcription levels of key metabolic factors that influence cell reactivation and floating before entering the water column.

This study showed that phycocyanobilin concentration in water increases gradually from January to May and reaches a maximum in May. The maximum concentration was maintained throughout winter. At N2, the phycocyanobilin concentration in sediments increased gradually from January to March and reached a maximum in March. This indicates that the deposited *Microcystis* community settled close to sediments. The structure of Microcystis colonies disintegrates throughout the depolymerisation of the *Microcystis* community and the individual cells then live through the winter in sediments in a vegetative state. Once the temperature rises, it receives proper illumination and its photosynthesis recovers immediately. Rich nutrients in the sediments are enough to cause algal growth, forms a new community and enters the water body, resulting in cyanobacterial bloom (Reynolds et al., 1981).

The transcription level of *gvpC* at N2 increased gradually and reached a maximum in March. This may have been due to the expression growth of *gvpC*, which results in the cyanobacteria synthesizing gas vesicles quickly, causing them to float to the surface. However, the expression level of *gvpC* declines gradually in the growth and floating stages of the cyanobacterial biomass. This may demonstrate that expression of *gvpC* and the formation of gas vesicles might be an indicator of reactivation and subsequent buoyancy of *Microcystis* and a continuous expression of *gvpC* is not required after algal cells revert to normal cells. These results conform to the experimental laboratory results in previous studies (Zhang et al., 2011): the relative contents of gas in gas vesicles increased first and then decreased and the transcription level of *gvpC* declined in the *Microcystis* during the floating process. The study has also pointed out that the gas vesicles are the main buoyancy source for *Microcystis*. This buoyancy can only make *Microcystis* buoyant within the water column, but is inadequate to make *Microcystis* float to the surface and form an algal bloom (Kromkamp, Botterweg & Mur, 1988).

Differing from *gvpC* expression, the relative expression of *mcyA* increased gradually before reaching a maximum rapidly. The relative expression of *espL* increased rapidly to start but then declined steadily. EPS, which is released by *Microcystis* cells, is the main component of the sheath of *Microcystis* and the sheath plays an important role in *Microcystis* floating during reactivation.

In this study, a SYBR Green qRT-PCR method was developed to detect the expression level of *gvpC*, *mcyA*, and *espL* in eutrophic Lakes. This method is characterized by a simplicity, quick detection, and high accuracy. Results demonstrated that the melt curve showed a single specificity peak and the amplification curve was in a typical S shape and had strong specificity. The qRT-PCR method is highly repeatable in repeated experiments between samples (*n* = 3). Detection of gene expression levels in field samples from Lake Taihu prove that the proposed method is feasible for detecting expression levels of *gvpC*, *mcyA*, and *espL*. RNA extraction concentration and purity were improved with the optimizations of Qiagen RNeasy Mini Kit and OMEGA Soil RNA Kit.

The results from Meiliang Bay and the center of Lake Taihu show that the *Microcystis* abundance and proportion, and the eutrophication rates were highest in Meiliang Bay compared to those in the center of Lake Taihu, indicating that the growth in nutrient concentration was conducive to the growth and maintenance of competitiveness of *Microcystis*. These results conform to field surveys and laboratory results of other studies. Expression of *gvpC* might be a transition indicator of the reactivation and buoyancy of *Microcystis*.

Algal toxins not only help *Microcystis* resist foreign predators, but also contribute to the formation of dominant species in *Microcystis*. Microcystic toxins released into the external environments of cells act as signal substances and can facilitate the formation of the *Microcystis* community by inducing release of EPS. Such *Microcystis* structures further intensifies its biomass within water column and is conducive to the formation of a dominant species. This study lays a foundation for understanding the formation of cyanobacterial blooms at the molecular, physiological, and ecological level, and offers more scientific data that may be used to develop control mechanisms for cyanobacterial blooms.

## Conclusions

This study showed that the qRT-PCR method was used to research quantitative detection on recruitment of cyanobacteria. The recruitment of cyanobacteria was the process in which cyanobacteria in the sediment began to regain their activity, started to grow and migrated to the water column. Nutrient levels within Lake Taihu are high, often causing serious blooms. Therefore, the process of studying the recruitment of cyanobacteria has theoretical and practical significance for predicting cyanobacterial blooms and taking timely measures to slow down the ecological disasters of cyanobacterial blooms.

## Supplemental Information

10.7717/peerj.7188/supp-1 Supplemental Information 1 Raw data: Relative expression of *gvpC,mcyA,espL* in RNA of cyanobacteria in water.

10.7717/peerj.7188/supp-2 Supplemental Information 2 Raw data: Relative expression of *gvpC,mcyA,espL* in RNA of cyanobacteria in sediments.

10.7717/peerj.7188/supp-3 Supplemental Information 3 Raw data: Phycocyanobilin concentration in water.

10.7717/peerj.7188/supp-4 Supplemental Information 4 Raw data: Phycocyanin concentration in sediments.

## Additional Information and Declarations

### Data Availability

The following information was supplied regarding data availability: Raw data are available in the Supplemental Files.
